# Dynamic Modeling and Simulation of a Body Weight Support System

**DOI:** 10.1155/2020/2802574

**Published:** 2020-02-12

**Authors:** Zhendong Song, Wei Chen, Wenbing Wang, Guoqing Zhang

**Affiliations:** ^1^Shen Zhen Polytechnic, 4089 Shahe West Road, Shenzhen 518055, China; ^2^People's Hospital of Gaoxin, 768 Fudong Road, Weifang 261205, China

## Abstract

This paper proposes a body weight support (BWS) system with a series elastic actuator (SEA) to facilitate walking assistance and motor relearning during gait rehabilitation. This system comprises the following: a mobile platform that ensures movement of the system on the ground, a BWS mechanism with an SEA that is capable of providing the desired unloading force, and a pelvic brace to smooth the pelvis motions. The control of the body weight support is realized by an active weight-offload method, and a dynamic model of the BWS system with offload mass of a human is conducted to simulate the control process and optimize the parameters. Preliminary results demonstrate that the BWS system can provide the desired support force and vertical motion of the pelvis.

## 1. Introduction

Based on the data of the World Health Organization in 2016, more than six million stroke survivors are disabled yearly [1]. Furthermore, stroke is a leading cause of serious long-term disability [2], and dyskinesia hinders gait training and rehabilitation [3]. A disability of the lower extremity limits functional independence in activities of daily living. Studies have shown that robot-assisted rehabilitation training is more effective than traditional gait training in improving walking ability in stroke patients [4], and body weight support (BWS) treadmill training is effective in enhancing patient mobility [5, 6]. During gait training, the pelvic vertical motion in the coronal plane plays an important role in the transformation between potential energy and kinetic energy, and hemiplegic patients need active mass-offloading to support their body weight. However, the guidance hypothesis in motor control research suggests that position-control-based movement might decrease motor learning for certain tasks [7]. Therefore, gait rehabilitation with flexible robots could help in providing the required dynamic force while also allowing for normal pelvic motions. Several robotic approaches have been developed to facilitate recovery based on the BWS system. Traditionally, an elastic spring [8], a winch [9], or a counterweight [10] with a wire harness is used in commercial BWS systems to relieve body weight.

**Figure 1:**
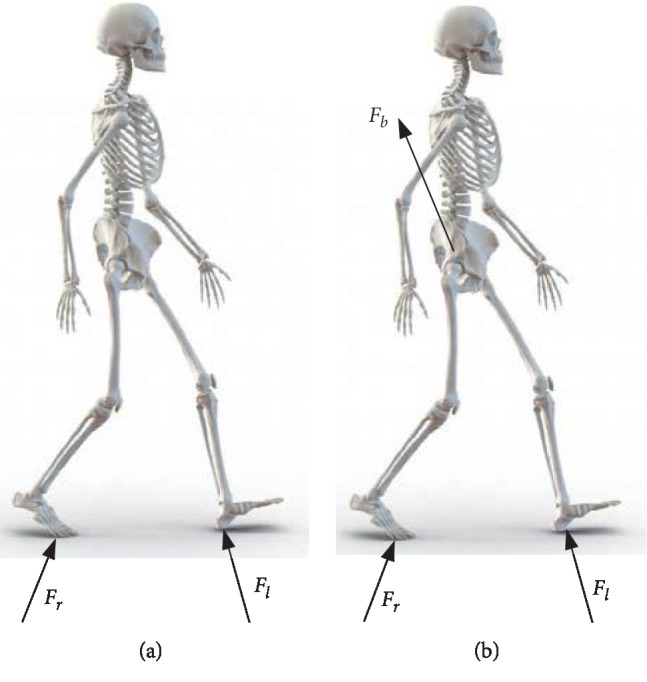
Forces acting on a human during gait: normal gait (a) without BWS and (b) with BWS.

However, the traditional BWS systems usually restrict the pelvis and trunk motion. Such a restriction is not beneficial to balance recovery. Thus, active BWS systems with pelvic support and novel control algorithm were studied as shown in Figure 1. A height-adjustable saddle was employed in the Pacer Gait Trainers of Rifton Equipment Co., Ltd. to assist gait training [11], and a pneumatic pusher was used to provide mass-offloading. GaitEnable [12], Robotic Walker [13], and Kineassist [14] can provide patients with active BWS and part of passive pelvic motions by using a linear actuator and pelvic brace. The balance assessment robot (BAR) with four linear actuators and interaction force sensors was developed by Olenšek et al. [15] to control the pelvic position and orientation. The BAR can monitor the support force and provide adjustments in real time. In addition, a wire-driven pelvic support mechanism, namely, tethered pelvic assist device (TPAD), was developed by Kang et al. [16]. The TPAD can guide the pelvic motions by controlling the interaction forces acting on the pelvis. However, the active BWS series elastic actuators (SEAs) are often considered to improve training safety. Thus, a pelvic support robot with BWS was developed and active BWS was provided for the vertical motion of the pelvis.

The rest of the paper is organized as follows.  Section 2 contains a description of the robot.  Section 3 describes the modeling and controller design of the system.  Section 4 presents the MATLAB/Simulink simulation and discussion. Finally, Section 5 provides conclusions and future work.

## 2. System Description

**Figure 2:**
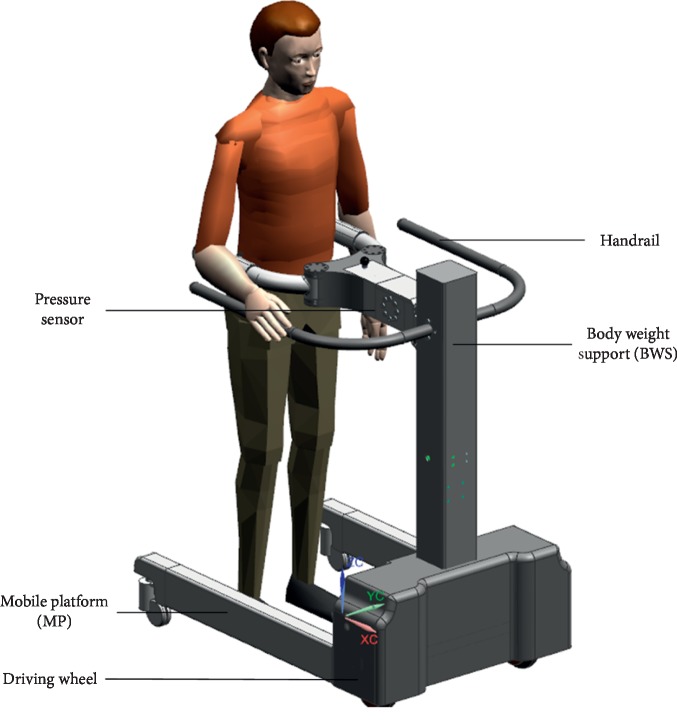
CAD model of the system.

A BWS system generally works in conjunction with a treadmill or a mobile platform (MP) to achieve gait training. Compared with the treadmill, the advantages of the MP are as follows: (1) the training environment is realistic, and the participants can feel the acceleration of the center of mass (CoM); (2) family rehabilitation training could be realized in this platform with a small MP. Therefore, a robot comprising a mobile platform, a BWS system, and a pelvic brace was designed as shown in Figure 2.

### 2.1. Robot Design

The overall conceptualized design and the prototype of the robot are shown in Figure 2. Three force sensors are used to identify the motion intention, and then the computer controls the motor of the BWS to support the body weight and two driving wheels to follow human motions. The modeling and control of the BWS are mainly discussed in this study.

The MP mainly aims at providing over-ground mobility, thereby achieving the gait rehabilitation training. The MP comprises two active wheels to provide power, two passive castors to maintain balance, and a U-shaped rigid steel frame. Based on the motions of the lower limbs, the U-shaped rigid steel frame is designed to satisfy approximately 0.8 and 1.2 m of free space in the medial/lateral and the anterior/posterior directions, respectively. The rotation of the robot is realized by the differential driving of the two wheels.

The BWS driven by a servo motor is designed to achieve the vertical displacement of the pelvis (approximately 0.2 m) and provide subjects with appropriate BWS via a guide screw and a set of linear guideways. A force sensor is installed to measure the interaction force between the robot and the human. A set with a ball spline and two springs is installed to achieve accurate force control and facilitate comfortable interaction with the robot. This part is used to reduce the undesirable forces generated by the control lag. The system control is implemented in TwinCAT2 using a controller (Beckhoff PLC CX5130). Based on the error between the desired and the interaction forces, the servo motor generates the corresponding dynamic force to support the human body weight.

A pelvic brace is designed to smooth the pelvic motions and install the force sensors. A pelvic brace is designed based on the range of pelvic motions during normal gait to achieve pelvic obliquity and rotation. A force sensor is installed between the BWS and the pelvic brace, and then a revolute pair is used to achieve pelvic obliquity. Afterward, a pair of cambered slideway is used to achieve pelvic rotation. At the end of the pelvic brace, two pressure sensors are installed, and the force signal is used to identify the motion intentions of the users.

### 2.2. Problem Statement

During bipedal walking with a treadmill, the interchange between kinetic energy and potential energy is a continuous process, and the vertical motion of the CoM is crucial in this process [17]. However, for the BWS, the vertical motion is unfavorable for the constant value of the mass-offloading, particularly for the passive BWS systems with counterweights, winches, or springs. Thus, the active BWS systems are developed to improve the offloading accuracy and dynamic performance of the BWS [18–22]. However, the existing rigid BWS systems usually generate undesirable forces and rigid impacts due to the existence of control lag and inertia. Meanwhile, the force-control-based strategies are used to provide users with appropriate force assistance to guide the pelvic movements. Thus, the SEA BWS is used to control the robot.

## 3. Modeling and Control

### 3.1. Dynamic Model

**Figure 3:**
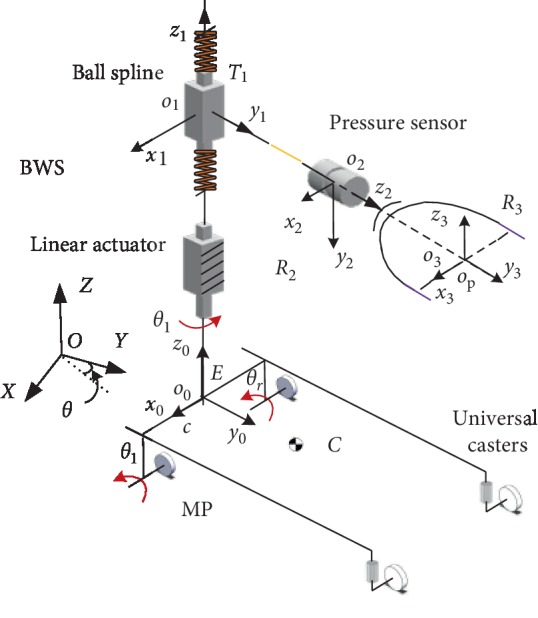
Schematic of the system.

The kinematics model of the rehabilitation robot is shown in Figure 3, where OXYZ is the global coordinate system, point *E* is the midpoint of two passive castors, and *o*_0_*x*_0_*y*_0_*z*_0_ is the local coordinate system attached to the robot. The BWS system is located at point *E*, through the pelvic brace connected to the pelvic center *O*_*p*_, and point *C* is the mass center of the rehabilitation robot. Using the position, orientation and velocity of point *E* to, respectively, indicate the position, orientation, and velocity of the robot, *θ* is the heading angle of the mobile platform relative to the *X*-axis. 2*c* is the distance between the two driving wheels, *a* is the distance from point *E* to pelvic center projected to *Oxy*, *r* is the radius of driving wheel, and *d* is the distance from the mass center *C* to the reference point *E*.

The velocity matrix of the MP relative to the global coordinate system is defined as $\dot{q}={\left[\begin{array}{ccc} \dot{x} & \dot{y} & \dot{\theta } \end{array}\right]}^{T}$, angular velocity of two driving wheels as $\omega ={\left[\begin{array}{cc} {\dot{\theta }}_{l} & {\dot{\theta }}_{r} \end{array}\right]}^{T}$, and the velocity matrix relative to a local coordinate system as ${\dot{q}}_{R}={\left[\begin{array}{ccc} {\dot{x}}_{R} & {\dot{y}}_{R} & {\dot{\theta }}_{R} \end{array}\right]}^{T}$. Under the circumstances that the kinestate of driving wheels is pure rolling without sliding and the mobile platform can perform instantaneous motion along heading direction of the driving wheels, kinematics equation of the robot can be obtained based on the following geostatistics:

(1) $$\begin{array}{c} {\dot{q}}_{R}=\left[\begin{array}{cc} \frac{r}{2} & \frac{r}{2}\\ 0 & 0\\ \frac{r}{2c} & -\frac{r}{2c} \end{array}\right]\left[\begin{array}{c} {\dot{\theta }}_{l}\\ {\dot{\theta }}_{r} \end{array}\right]. \end{array}$$

By taking point *E* as a reference point, equation (5) premultiplies the rotation matrix and ${\dot{q}}_{R}$ translates to robot velocity in a global coordinate system:

(2) $$\begin{array}{c} \dot{q}=\left[\begin{array}{ccc} \cos\;\theta & 0 & -e\;\sin\;\theta \\ \sin\;\theta & 0 & e\;\cos\;\theta \\ 0 & 0 & 1 \end{array}\right]{\dot{q}}_{R}. \end{array}$$

For the control of the MP, speed control is used to follow the motion on the ground. Meanwhile, torque control is utilized to control the BWS system to provide accurate offloading for users. Thus, the dynamic model is calculated.

**Figure 4:**
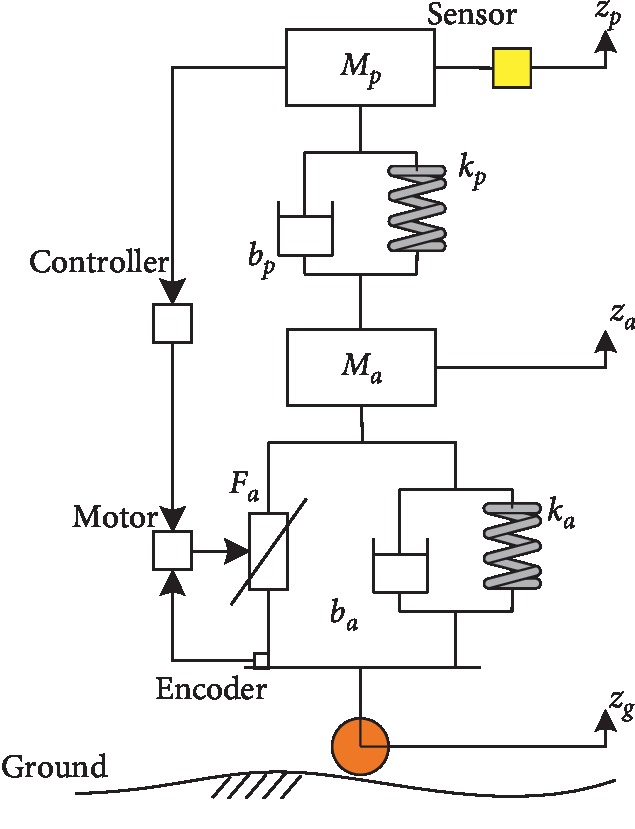
Mass-spring-damper system of the BWS.

The estimated value of the sensor is used to replace the actual value, to put the theoretical model into a simulated program as shown in Figure 4, and then the interaction force can be expressed as follows:

(3) $$\begin{array}{c} F_{h}=\frac{\left(k_{s}\theta_{s}\right)}{l}, \end{array}$$

where *k*_*s*_ is the elastic coefficient of the torque sensor, $l=\left\|\vec{o_{s}P_{0}}\right\|$ is the distance from the sensor to the pelvic center, and *θ*_*s*_ is the deformation of the sensor, which can be expressed as follows:

(4) $$\begin{array}{c} \theta_{s}=2\;\sin^{-1}\left(\frac{z_{h}-z_{p}}{2l}\right), \end{array}$$

where *z*_*h*_ is the position of pelvic center and *z*_*p*_ is the *z*-axis component of the mass center of *M*_*p*_, that is, the center of the ball spline. Based on the Newton–Euler method, the equilibrium equation of *M*_*p*_ can be expressed as follows:

(5) $$\begin{array}{c} F_{h}+b_{p}\left({\dot{z}}_{p}-{\dot{z}}_{a}\right)+k_{p}\left(z_{p}-z_{a}\right)+M_{p}\;g+M_{h}g=M_{p}{\ddot{z}}_{p}, \end{array}$$

where *b*_*p*_ is the viscosity coefficient of the ball spline, *k*_*p*_ is the elastic coefficient of the spring, *M*_*h*_ is the mass of the pelvic brace, *z*_*a*_ is the *z*-axis component position of the guide screw, ${\dot{z}}_{a}$ is the velocity of the guide screw, ${\dot{z}}_{p}$ is the velocity of the ball spline, ${\ddot{z}}_{p}$ is the acceleration of the ball spline, and *g* is the gravitational constant. Then, ${\ddot{z}}_{p}$ can be obtained as follows:

(6) $$\begin{array}{c} {\ddot{z}}_{p}=\frac{F_{h}+b_{p}\left({\dot{z}}_{p}-{\dot{z}}_{a}\right)+k_{p}\left(z_{p}-z_{a}\right)+M_{p}g+M_{h}g}{M_{p}}. \end{array}$$

The equilibrium equation of *M*_*a*_ for the guide screw can be expressed as follows:

(7) $$\begin{array}{c} F_{a}+b_{p}\left({\dot{z}}_{p}-{\dot{z}}_{a}\right)+k_{p}\left(z_{p}-z_{a}\right)+b_{a}\left({\dot{z}}_{a}-{\dot{z}}_{g}\right)+k_{a}\left(z_{a}-z_{g}\right)+M_{a}g=M_{a}{\ddot{z}}_{a}, \end{array}$$

where *F*_*a*_ is the desired force, *b*_*a*_ is the viscosity coefficient of the guide screw, *k*_*a*_ is the elastic coefficient of the guide screw, *z*_*g*_ is the *z*-axis component of the driving wheel, ${\dot{z}}_{g}$ is the velocity of the driving wheel, and ${\ddot{z}}_{a}$ is the acceleration of the guide screw. Then, ${\ddot{z}}_{a}$ can be obtained as follows:

(8) $$\begin{array}{c} {\ddot{z}}_{a}=\frac{F_{a}+b_{p}\left({\dot{z}}_{p}-{\dot{z}}_{a}\right)+k_{p}\left(z_{p}-z_{a}\right)+b_{a}\left({\dot{z}}_{a}-{\dot{z}}_{g}\right)+k_{a}\left(z_{a}-z_{g}\right)+M_{a}g}{M_{a}}. \end{array}$$

By combining equations (3)–(7), the dynamic equation of the BWS system can be obtained as follows:

(9) $$\begin{array}{c} \tau_{c}=M\ddot{q}+B\left(q,\dot{q}\right)+J^{T}F_{v}, \end{array}$$

where *τ*_*c*_ is the desired torque of motor; *M* is the generalized mass; $B\left(q,\dot{q}\right)$ is the generalized Coriolis, centrifugal, and external forces, including the gravity forces; $\ddot{q}$ is the generalized acceleration; *F*_*v*_ is the offloading; and *J*^*T*^ is the configuration-dependent Jacobian matrix. The transformation relationship from the desired force to the desired torque can be expressed as follows:

(10) $$\begin{array}{c} \tau_{c}=\frac{F_{a}L}{2\pi \eta }+I_{m}{\ddot{\theta }}_{m}, \end{array}$$

where *L* is the helical pitch of the guide screw, *η* is the transmission efficiency, *I*_*m*_ is the inertia of the motor, and ${\ddot{\theta }}_{m}$ is the angular acceleration of the motor.

### 3.2. Torque Control

**Figure 5:**
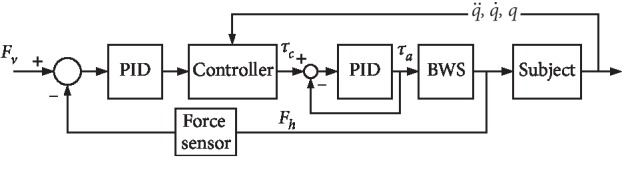
Control architecture of the system.

The control algorithm of the robot is divided into two control levels: (1) high-level controller and (2) low-level controller. The task of the high-level controller is to recognize the motion intention of the user.  Figure 5 shows that the task of the low-level controller is to filter the input signals and output the appropriate torque. The low-level and high-level controllers are executed at 1000 and 100 Hz, respectively.

The goal of the torque controller is to provide the user with stable mass-offloading, and the control architecture of the BWS system is shown in Figure 5. *F*_*v*_ is the desired value of offloading, and *F*_*h*_ is the input signal from the torque sensor, that is, the interaction force. When the user walks on the ground, the vertical motion of the pelvis will result in the change in input signal, and then the controller will generate torque to decrease the error between the desired and interaction forces. The torque controller with double-layer PID and the dynamic term is then given by the following equation:

(11) $$\begin{array}{c} e=F_{v}-F_{h},\\ \dot{e}={\dot{F}}_{v}-{\dot{F}}_{h},\\ \tau_{a}=M\ddot{q}+B\left(q,\dot{q}\right)+J^{T}F_{v}+K_{p}e+K_{d}\dot{e}+K_{i}\int e\text{ d}t, \end{array}$$

where *e* is the error between desired offloading and observed force, and the gain matrices for the first layer PID controller are provided by *K*_*p*_, *K*_*d*_, and *K*_*i*_. The goal of the second layer PID is to ensure that the output torque is coincident with the desired torque.

## 4. Simulation and Verification

Simulation studies were conducted in this paper to verify the effectiveness of the proposed control method. The MATLAB/Simulink was used in the simulation to simulate the processes of the BWS with the proposed method.

### 4.1. Simulation

**Figure 6:**
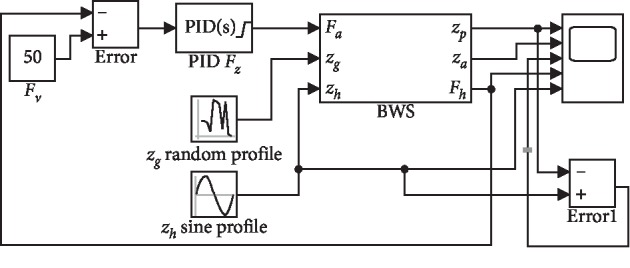
Simulation block diagram in MATLAB/Simulink.

**Figure 7:**
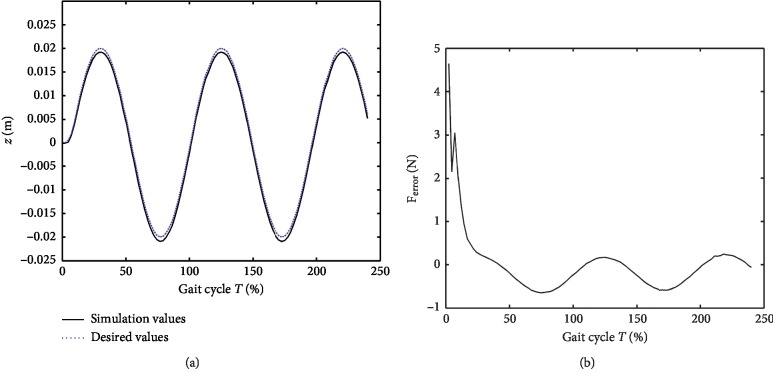
Simulation results with 0 kg offloading. (a) Vertical position of pelvic center. (b) Force error.

**Figure 8:**
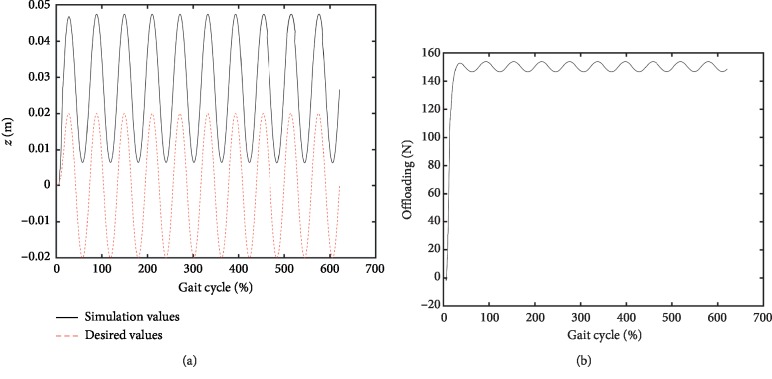
Simulation results with 15 kg offloading. (a) Vertical position of pelvic center. (b) Offloading force.

The simulation block diagram is shown in Figure 6. In the simulation process, the *z*-axis component of the driving wheel (*z*_*g*_) was a random profile block, and the vertical motion of the pelvis (*z*_*h*_) was assumed as a sine profile block. The “BWS” block was the compute module based on equations (3)–(7), and the solver was ode45. Two different cases of offloading, namely, 0.0 N and 150.0 N, were simulated in this paper. The parameters of the PID were adjusted during the simulation to achieve the desired characteristics. The conditions of the aforementioned trials are summarized in Table 1. The simulation results are shown in Figures 7 and 8. The simulation values, desired values, and the error between the simulation and desired forces for the case of 0.0 N offloading are given in Figure 7. Meanwhile, the simulation values, desired values, and the simulation force for the case of 150.0 N offloading are given in Figure 8.

As shown in Figure 7, the controller updated the desired torque during the gait cycle to follow the position of the pelvic center, while the torque controller was used to ensure the mass-offloading. The changed simulation values resembled the desired values with small steady-state error during the simulation, and the steady-state error of offloading was less than 2 N. This phenomenon demonstrated the capability to track dynamical parameters. Figure 8 shows that the controller was used to track the position of the pelvic center and provide 150.0 N offloading during the gait cycle. The changed simulation values resembled the desired values during the simulation, but the position error was approximately 0.025 m mainly due to the stiffness of the force sensor. The mean error of offloading was less than 3.1 N, demonstrating the capability to track dynamical parameters.

### 4.2. Discussion

The simulation results show that the proposed method was capable of providing the desired mass-offloading with small steady-state error, but the position tracking must consider the stiffness of the force sensor. Less time is needed to achieve a stable state when using the PID controller. Furthermore, the parameters of the PID must adjust to achieve the desired characteristics with different cases of the mass-offloading. The system inertia is increased with the mass-offloading. Therefore, the PID parameters must be adjusted to provide the desired mass-offloading.

## 5. Conclusion

This paper introduces a novel robotic BWS device for disabled people to conduct rehabilitation training. The present work also demonstrates the potential capability of a novel BWS system. Dynamic modeling and control algorithms are proposed based on the design concept to optimize the control of the interaction force. The preliminary simulation verification with MATLAB simulation is conducted to prove the effectiveness of the control algorithm. The BWS system may be used to improve the pelvic control function of stroke survivors. Furthermore, the robot will be fabricated, and the control algorithm of mass-offloading will be studied in the future.

## Figures and Tables

**Table 1:** Inertial properties of links.

Link	Masses (kg)	Position vector of CG (m)
1 (T)	50	0.55
2 (R)	8	0.15
3 (R)	15	0.2

## Data Availability

The simulation results and experimental results data used to support the findings of this study are available from the corresponding author upon request.
